# Forbs enhance productivity of unfertilised grass-clover leys and support low-carbon bioenergy

**DOI:** 10.1038/s41598-017-01632-4

**Published:** 2017-05-02

**Authors:** Wen-Feng Cong, Jingying Jing, Jim Rasmussen, Karen Søegaard, Jørgen Eriksen

**Affiliations:** Aarhus University, Department of Agroecology, Tjele, 8830 Denmark

## Abstract

Intensively managed grasslands are dominated by highly productive grass-clover mixtures. Increasing crop diversity by inclusion of competitive forbs may enhance biomass production and sustainable biofuel production. Here we examined if one or all of three forbs (chicory, *Cichorium intybus* L.; caraway, *Carum carvi* L.; plantain, *Plantago lanceolata* L.) included in ryegrass-red clover mixtures enhanced above- and below-ground productivity, and assessed their biofuel potentials, based on a three-year experiment with and without fertilisation as cattle slurry. We determined herbage yield, standing root biomass, and estimated methane energy output and greenhouse gas (GHG) emissions per energy unit using life cycle assessment. Results showed that plantain-containing grass-clover mixtures significantly increased herbage yield, while chicory- or caraway-containing mixtures maintained similar yields to the grass-clover mixture. Standing root biomass of the grass-clover mixture was enhanced by inclusion of caraway and plantain, with that of plantain further enhanced by fertilisation. The highest methane energy output was achieved in plantain-containing grass-clover mixtures. All unfertilised mixtures achieved the 60% reduction in GHG emissions compared to fossil fuel, whereas all fertilised mixtures did not meet the 60% reduction target. These findings suggest that including competitive forbs such as plantain in grass-clover mixtures enhances productivity, supporting low-carbon footprint bioenergy production.

## Introduction

Increasing global population poses huge challenges in sustainable production of food, feed and fuel, meanwhile reducing anthropogenic impacts on climate, environment and biodiversity^1^. This has among other things led to an increased replacement of fossil fuel with renewable energy sources e.g. bioenergy. Global bioenergy production is projected to further increase, e.g. the International Energy Agency estimates that up to 27% of the world transportation fuels will be covered by biofuels in 2050^2^. Such a transition should, however, be done in a way where bioenergy production is integrated in the land use for food and feed production. Furthermore, bioenergy production should markedly reduce greenhouse gas (GHG) emissions, which the EU’s Renewable Energy Directive sets a target of at least 60% reduction in GHG emissions compared to fossil fuel^3^. Hence, development of low input – high output – low carbon (C) footprint biomass crops is urgently needed to address these challenges.

Perennial legume-based grasslands, such as perennial ryegrass-white or red clover mixtures are widely used in intensively managed grasslands due to their high biomass production, efficient use of resources in temporal or spatial niches, and the addition of nitrogen (N) in the system via symbiotic N_2_ fixation largely replacing synthetic N fertiliser^4–6^. Moreover, incorporating the perennial legume-based grasslands in the crop rotations could be a means of improving soil fertility for subsequent arable crops^7^. Yet, these intensively managed grasslands are characterized by a limited number of plant species. Increasing diversity of plant species and/or functional groups is often found to increase above- and below-ground biomass production in natural grasslands, mainly through complementary use of resources^8–11^. This has raised an interest in investigating whether increasing species diversity can also enhance productivity of intensively managed grasslands. Agronomists have recently identified three productive deep-rooting perennial forbs: chicory (*Cichorium intybus* L.), caraway (*Carum carvi* L.) and plantain (*Plantago lanceolata* L.)^12–15^. These deep-rooting forbs are known to complementarily utilize resources (e.g. light and N) in shallow-rooted grass-clover mixtures^16^, holding the potential to further enhance the productivity of intensively managed grasslands. But it is yet to be elucidated if the inclusion of forbs leads to greater above- and below-ground primary production, and thus greater bioenergy production, soil C sequestration and lower GHG emissions.

To date, the growing of diversified perennial legume-based grasslands for bioenergy production has mainly taken place on marginal land with a low yield potential^17–19^, because bioenergy cropping in such areas competes little or not at all with food or feed production. Recent agricultural statistics show, however, that most developed countries are witnessing increasing areas with surplus agricultural grassland (e.g. predicted to be 13–22% of the permanent grassland in Europe by 2020), because of improved animal performance^20^. These surplus agriculturally managed grasslands are generally more fertile than marginal land, representing a substantial bioenergy production potential.

The most common way of using grassland biomass for bioenergy production in Europe is to convert harvested biomass into methane through anaerobic digestion^20^. Management practices such as fertilisation generally increase biomass production in managed grasslands and thus enhance methane energy output^21, 22^, whereas fertilisation also requires a higher energy input and contributes to higher GHG emissions related to, e.g., nitrous oxide (N_2_O) emissions derived from production and field application of N fertiliser^23^. Hence, it is necessary to systematically assess the efficiency of bioenergy production and estimate total GHG emissions throughout the cycle of energy production, including not least the cultivation step which is of primary importance^19, 23^.

Here we studied the impacts of including three forbs in fertilised and unfertilised grass-clover mixtures on above-and below-ground productivity, bioenergy potential and climate impact. We employed a three-year field experiment to determine annual yield and standing root biomass, and combined these data with a life cycle assessment to estimate net energy balance and GHG emissions per energy unit. We hypothesized that: (1) inclusion of competitive forbs in the grass-clover mixture would improve productivity and bioenergy production, and (2) that unfertilised mixtures would have greater potential for GHG mitigation than fertilised mixtures due to field N_2_O emissions from fertilisation.

## Methods

### Experimental site

The experiment was established in spring 2013 in the long-term (since 1987) organic dairy crop rotation at the Foulumgaard Experimental Station, Aarhus University, Denmark (56°29′44 N, 9°34′3 E)^24^. The preceding crop in 2012 was winter rye. The experimental site was situated on a loamy sandy soil (8% clay, 10% silt and 82% sand), developed on moraine material from the Weichselian glacial age. The soil had a pH of 5.9, and contained 2.0% organic C and 0.17% total N. In the study years 2013, 2014 and 2015, the annual mean temperature was 7.8, 9.5 and 8.6 °C, respectively, while annual precipitation was 632, 853 and 904 mm, respectively.

### Experimental treatments and management

The grassland experiment was laid out as a completely randomized block design with three replicates. In each of the three blocks, experimental treatments were arranged in a 15 × 2 factorial design with species composition and fertilisation as the two fixed factors. The 15 levels of sown species composition consisted of five pure stands (perennial ryegrass, *Lolium perenne* L.; red clover, *Trifolium pratense* L.; chicory, *Cichorium intybus* L.; caraway, *Carum carvi* L.; ribwort plantain, *Plantago lanceolata* L.) and 10 seed mixtures of these species with different seed proportions (Table 1). Two levels of fertilisation in the form of cattle slurry, 0 and 250 kg total N ha^−1^ yr^−1^ (52% NH_4_-N), were applied across the species composition treatments. In total, we had 90 plots with the plot size of 8 m length × 1.5 m width. In each plot, 10 rows of seeds (0.12 m row distance) were sown by machine in May 2013. Adjacent plots were separated by a 0.3 m buffer. Based on previous empirical knowledge, optimum seed rates in pure stands were 15, 4 and 12 kg ha^−1^ for perennial ryegrass, red clover and the three forbs, respectively. The seed rate of each species in a mixture was calculated by multiplying the seed rate of the species in its pure stand with the proportion of the seed in the respective mixture (Table 1). Cattle slurry was applied four times during the growing season in both 2014 and 2015; with 100 kg N ha^−1^ yr^−1^ at the beginning of the growing season and 50 kg N ha^−1^ yr^−1^ immediately after the first, second and third harvests. All plots received a one-off 200 kg K ha^−1^ (K_2_SO_4_) application in May 2015 to avoid potassium deficiency. In dry periods of the growing seasons, 50–60 mm irrigation was applied in each plot to avoid water deficiency.

**Table 1:** Plant species and seed proportions in the 10 seed mixtures.

Seed mixtures	Ryegrass	Red clover	Chicory	Caraway	Plantain
Grass-clover^†^	50	50			
20% chicory	40	40	20		
20% caraway	40	40		20	
20% plantain	40	40			20
60% chicory	20	20	60		
60% caraway^†^	20	20		60	
60% plantain^†^	20	20			60
20% all forbs	40	40	6.7	6.7	6.7
60% all forbs^†^	20	20	20	20	20
80% all forbs	10	10	26.7	26.7	26.7

Numbers in the table referes to the proportions (%) of optimal seeding rate of plant species in pure stands. ^†^Four mixtures were selected for root sampling.

### Herbage dry matter yield

A plot harvester (Haldrup C-85, Denmark) was used to cut the herbage at 7 cm stubble height and record the fresh weight of the harvested biomass. The harvests were performed two times in 2013 (early July and early October) and four times in both 2014 and 2015 (late May, early July, mid-August and early October). In each harvest, a 500 g subsample was dried at 80 °C and weighed to calculate herbage dry matter (DM) yield. Botanical composition of the mixtures was determined by separating a 150–350 g subsample into the five sown species and unsown species. In the establishment year (2013) unsown species contributed more than 60% to the total DM yield and further quantitative analyses were made only with the more consolidated yields from 2014 and 2015. The DM yields of the five monocultures were used to calculate expected yields of the grass-clover mixture or forb-containing grass-clover mixtures based upon the proportion of seed mixtures. The difference between observed yield and expected yield of a specific mixture indicates overyielding.

### Standing root biomass

The auger method was employed to quantify standing root biomass^25^. Given that the forb-containing grass-clover mixtures contain both species with large taproots (e.g. red clover) and species with fibrous roots (e.g. ryegrass), a specific sampling strategy was developed to quantify root biomass by combining targeted in-row sampling of each plant species (stratified sampling) and random between-row sampling of fibrous and secondary roots (random sampling). The root sampling was conducted in late August 2015 (the final year) in 24 selected plots (two fertilisation levels × four species mixtures × three replicates). The selected four species mixtures were the grass-clover mixture, the grass-clover mixtures with 60% plantain, 60% caraway and 60% of all three forbs (see Table 1). We did not select the grass-clover mixture with 60% chicory because chicory almost vanished from the experimental plots in 2015, both in the pure stand and in seed mixture treatments.

**Figure 1:**
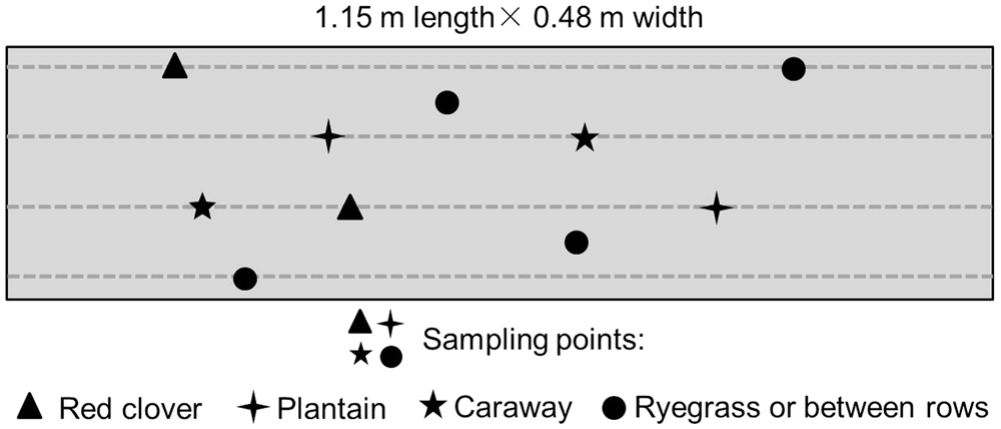
Root sampling in August in the grassland experiment. It is illustrated with a perennial ryegrass-red clover mixture including plantain and caraway. Dashed horizontal lines represent crop rows and symbols refer to sampling points for the different plant species (*n* = 2 for each species and for between-row samples). For red clover, caraway and plantain, each soil core (0–50 cm) for root sampling was taken on an individual plant.

Prior to the third harvest in mid-August (2015), a representative sampling area (1.15 m length × 0.48 m width) comprising four rows of plants was chosen in the central part of each plot (Fig. 1). Within the sampling area, the number of individual plants of red clover and forb species was counted. Two representative plants of each of the red clover and forb species were carefully chosen and marked for subsequent root sampling. Immediately after the harvest, soil cores (5 cm inner-diameter) were taken on the selected plants to capture the whole taproots with most of the root biomass within the core. Four additional soil cores were taken with two on ryegrass within rows and two between rows where root biomass mainly consisted of fibrous or secondary roots (Fig. 1). Thus, there were six cores in the grass-clover mixture (i.e. two cores on red clover, two cores on ryegrass and two cores between rows), eight cores in the mixtures with 60% plantain or caraway, and ten cores in the mixtures with 60% of all three forbs (i.e. not including cores for the vanishing chicory). Each core was subsequently divided into three depths (0–10, 10–20 and 20–50 cm), put into plastic bags and stored at 2 °C prior to washing (576 samples in total). Soil adhering to the roots was removed by washing the roots on a 425-µm sieve and by separating the roots from soil particles > 425 µm (e.g. sand) by repeated decantation from a container onto the sieve. Roots were dried at 70 °C for 48 h and weighed.

Root biomass (RB) at a specific soil depth in the sampling area was calculated as follows:

1 $$R{B}_{i}=(a\ast R{B}_{i,RC}+b\ast R{B}_{i,CA}+c\ast R{B}_{i,PL})+((R{B}_{i,f}/\pi {r}^{2})\ast (0.552-a\ast \pi {r}^{2}-b\ast \pi {r}^{2}-c\ast \pi {r}^{2}))$$

where ${{RB}}_{i}$ is the total root biomass (g m^−3^) at soil depth interval *i* in the sampling area (0.552 m^2^); *a*, *b*, *c* are the numbers of red clover, caraway and plantain plants, respectively, counted in the sampling area (Fig. 1); *RB*_*i,RC*_, *RB*_*i,CA*_ and *RB*_*i,PL*_ is, respectively, the root biomass (g m^−3^) within the soil core beneath red clover, caraway and plantain at soil depth interval *i*; *RB*_*i,f*_ is the root biomass (g m^−3^) averaged across the four cores taken on ryegrass within rows and between rows at soil depth interval *i*; and *r* is the radius of soil cores (i.e. 0.025 m). In summary, the first part of equation (1) was used to calculate taproot-dominated root biomass, while the final part represented the calculation of root biomass that was dominated by fibrous roots. Total root biomass for each treatment was finally extrapolated from the sampling area (0.552 m^2^) to a 1 m^2^ area.

### Bioenergy production and climate impact

We used the production process model of Carlsson, *et al*.^19^ to estimate the effect of species composition and fertilisation on bioenergy production and climate impact when using the harvested biomass as a substrate for biogas vehicle fuel. This model defined the system boundary including cultivation (ploughing, harrowing, fertilisation of cattle slurry and potassium, liming, soil compaction, sowing, rolling), harvesting (cutting and chopping), transportation, silage storage (compaction into silo and ensiling), feeding into biogas plant, biogas production, and subsequent upgrading of biogas to vehicle fuel quality (98% methane) and compression. Bioenergy production was then estimated by calculating the net energy balance, i.e., subtracting total energy input from methane energy output related to the whole production process. Methane energy output was calculated based on the harvested biomass and previously determined specific methane yields of grass-clover mixture and pure stands of chicory, caraway and plantain^26^. Energy input related to each of above-mentioned operational steps was calculated by employing field measured data or country-specific values^27, 28^ where possible; otherwise the IPCC^29^ default values were used. Climate impact was assessed by dividing total GHG emissions by the net energy balance, expressed as kg carbon dioxide (CO_2_) equivalents per gigajoule (GJ) of vehicle fuel (kg CO_2_-eq GJ^−1^). The latter was then related to the EU sustainability criteria for biofuel requiring a 60% emission reduction from 1 January 2018 onwards compared to fossil fuel^3^. Total GHG emissions were considered from five main processes, i.e. diesel consumption, production of lime and potassium fertilizer, silage storage, losses during biogas production and field N_2_O emissions from crop residues and cattle slurry. Notably, field N_2_O emissions from crop residues as a potentially large contributor to total GHG emissions may vary much between mixtures due to varying species composition and different N contents of above- and below-ground residues as well as below-ground to above-ground ratio. Therefore, the treatment-specific crop residue N measured directly from this study was used to estimate N_2_O emissions. For detailed calculations of the net energy balance and GHG emissions, please see Supplementary Text and Tables S1 and S2.

### Statistical analysis

Annual total DM yield of mixtures was statistically analysed by using a linear mixed-effects model with blocks as random effects and with species composition, fertilisation and study years as fixed effects. The linear mixed-effects model was also used to analyse total DM yield of mixtures and DM yield of component species for each study year, and standing root biomass with blocks as random effects and with species composition and fertilisation as fixed effects. Differences between factor levels (*n* > 2) were tested using Tukey’s *post hoc* test. All analyses were performed using the *R* software version 3.2.2.

## Results

### Effects of species composition and fertilisation on annual biomass yield

**Figure 2:**
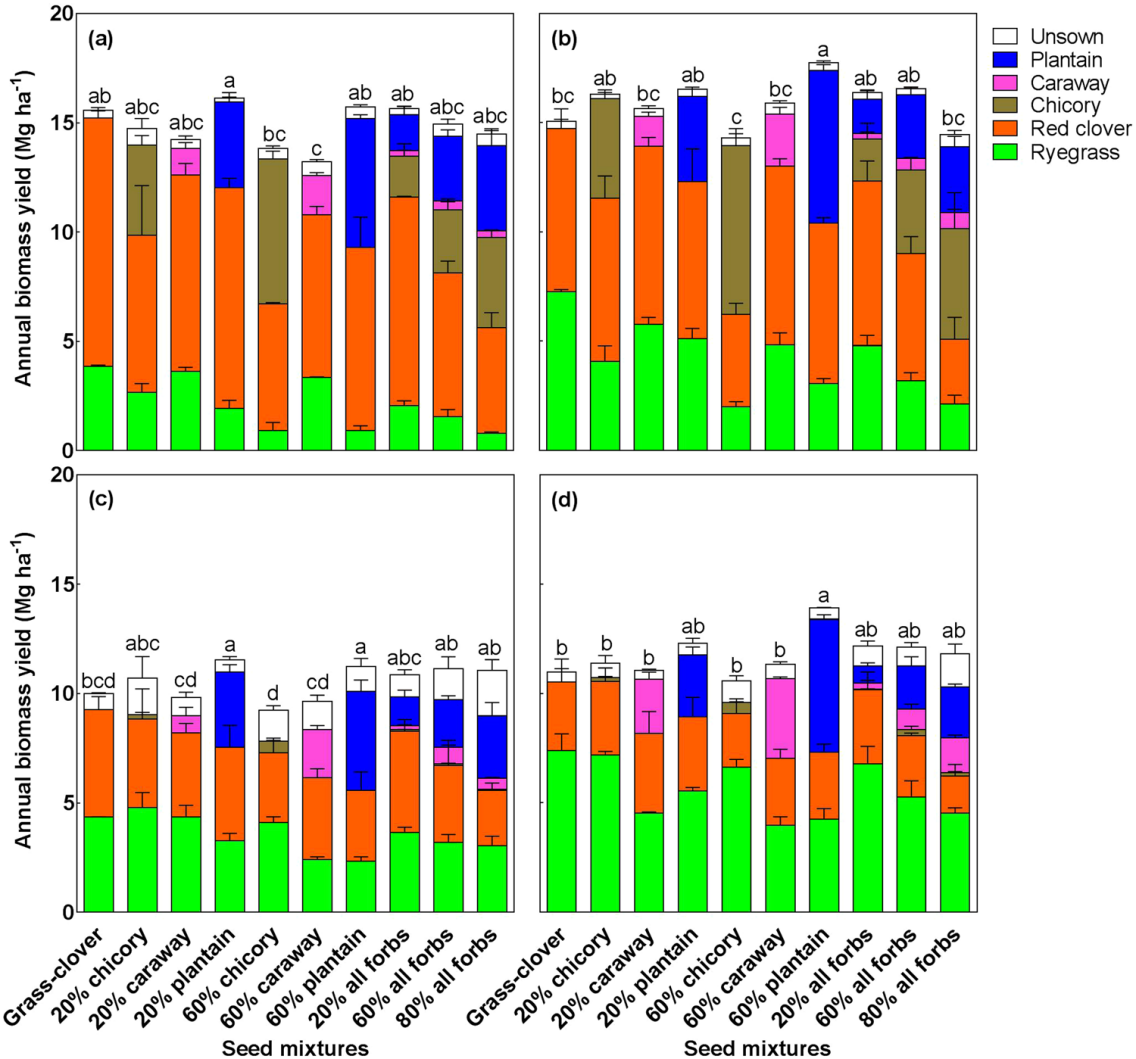
Annual biomass yield in the 10 seed mixtures in 2014 (**a**,**b**) and 2015 (**c**,**d**) without (**a**,**c**) and with (**b**,**d**) fertilisation. Data are means ± SE (*n* = 3) for each component species. Means for seed mixtures with different lowercase letters are significantly different (*P* < 0.05) using Tukey’s *post hoc* test.

Annual total DM yield significantly varied in the 10 seed mixtures (*P* < 0.001), with the differences consistent across two years (Species composition × Year: *P* = 0.13) and across the two fertilisation levels (Species composition × Fertilisation: *P* = 0.15) (Fig. 2). Inclusion of 60% plantain in the grass-clover mixture increased total DM yield by on average 14% compared to the grass-clover mixture (*P* < 0.01), while inclusion of 20% plantain increased DM yield by 10% (*P* = 0.04). Most other seed mixtures containing varying seeding proportions of chicory, caraway, or containing all three forbs produced statistically similar DM yields to the grass-clover mixture (Fig. 2). The annual DM yield of red clover in 2014 was significantly higher in the mixture with 60% plantain than in the mixture with 60% chicory, resulting in the significant difference in total DM yield between the two mixtures (Fig. 2a,b). Consistently higher biomass of plantain than caraway across the two years led to higher total DM yield in the mixture with 60% plantain than with 60% caraway (Fig. 2). The red clover yields across mixtures were significantly lower in 2015 than in 2014 (*P* < 0.001). The fraction of unsown species varied from 3% in 2014 to 9% in 2015, but did not significantly differ among the 10 seed mixtures across the two years. White clover was the dominant unsown species in this study, constituting more than 90% of unsown biomass.

Fertilisation significantly enhanced total DM yield (Mg ha^−1^) on average from 14.8 to 15.9 in 2014 and from 10.5 to 11.8 in 2015 (Fig. 2). Fertilisation consistently promoted the growth of ryegrass (*P* < 0.001), but suppressed the growth of red clover (*P* < 0.001) across the two years. Yields of the three forbs tended to slightly increase under fertilisation, although statistically insignificant (*P* > 0.05).

**Figure 3:**
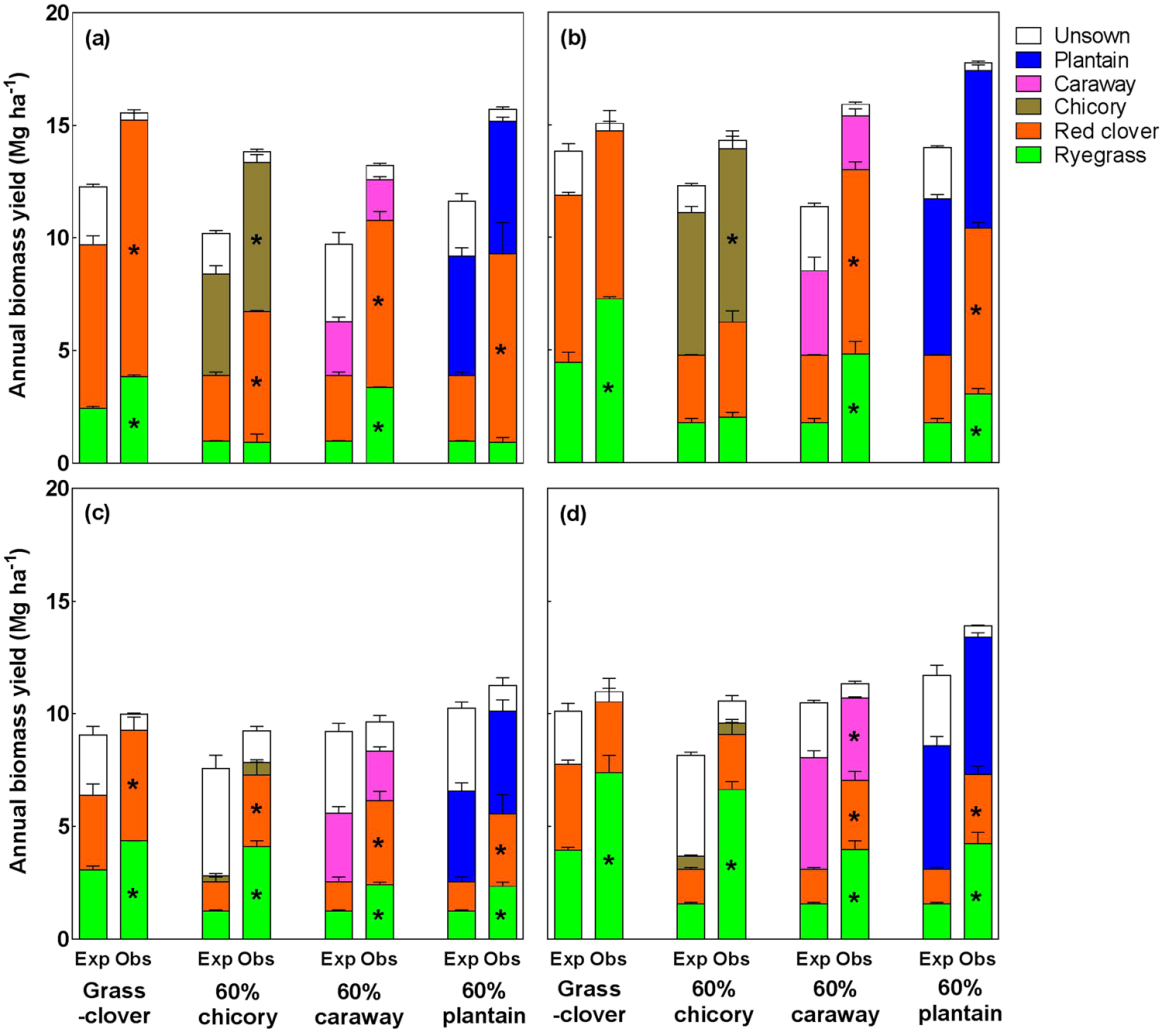
Annual biomass yield as observed (Obs) and expected (Exp) in the four seed mixtures in 2014 (**a**,**b**) and 2015 (**c**,**d**) without (**a**,**c**) and with (**b**,**d**) fertilisation. The expected values are calculated as the weighted mean biomass yield in pure stands in the respective year. Data are means ± SE (*n* = 3) for each component species. Asterisks refer to significant differences (*P* < 0.05) between expected and observed yield for each component species.

To indicate possible synergies between plant species, the total DM yields of the grass-clover mixture and three 60% forb-containing mixtures were compared with the expected values calculated from their respective pure stands (see Supplementary Figure S1). In the unfertilised grass-clover mixture, ryegrass and red clover overyielded by 50% and 53%, respectively, compared to expected values calculated from their respective pure stands, but the overyielding of red clover disappeared with fertilisation (Fig. 3). In the forb-containing mixtures, the performance of the forbs and their effects on ryegrass and red clover differed with forb species. Plantain had similar yields in its mixture and in its pure stand, but it consistently enhanced the growth of red clover by 145% averaged across the two years and two fertilisation levels. Caraway tended to underyield in its mixture compared to its pure stand, while it consistently promoted the growth of both ryegrass (+166%) and red clover (+153%). Chicory had a significantly higher yield in its mixture than in the pure stand in 2014, but it promoted the growth of red clover to a lesser extent (+71%) than plantain and caraway and did not affect the growth of ryegrass. Chicory almost vanished from the experimental plots in 2015, both in the pure stand (Supplementary Figure S1) and mixture plots (Fig. 2).

### Effects of species composition and fertilisation on standing root biomass

**Figure 4:**
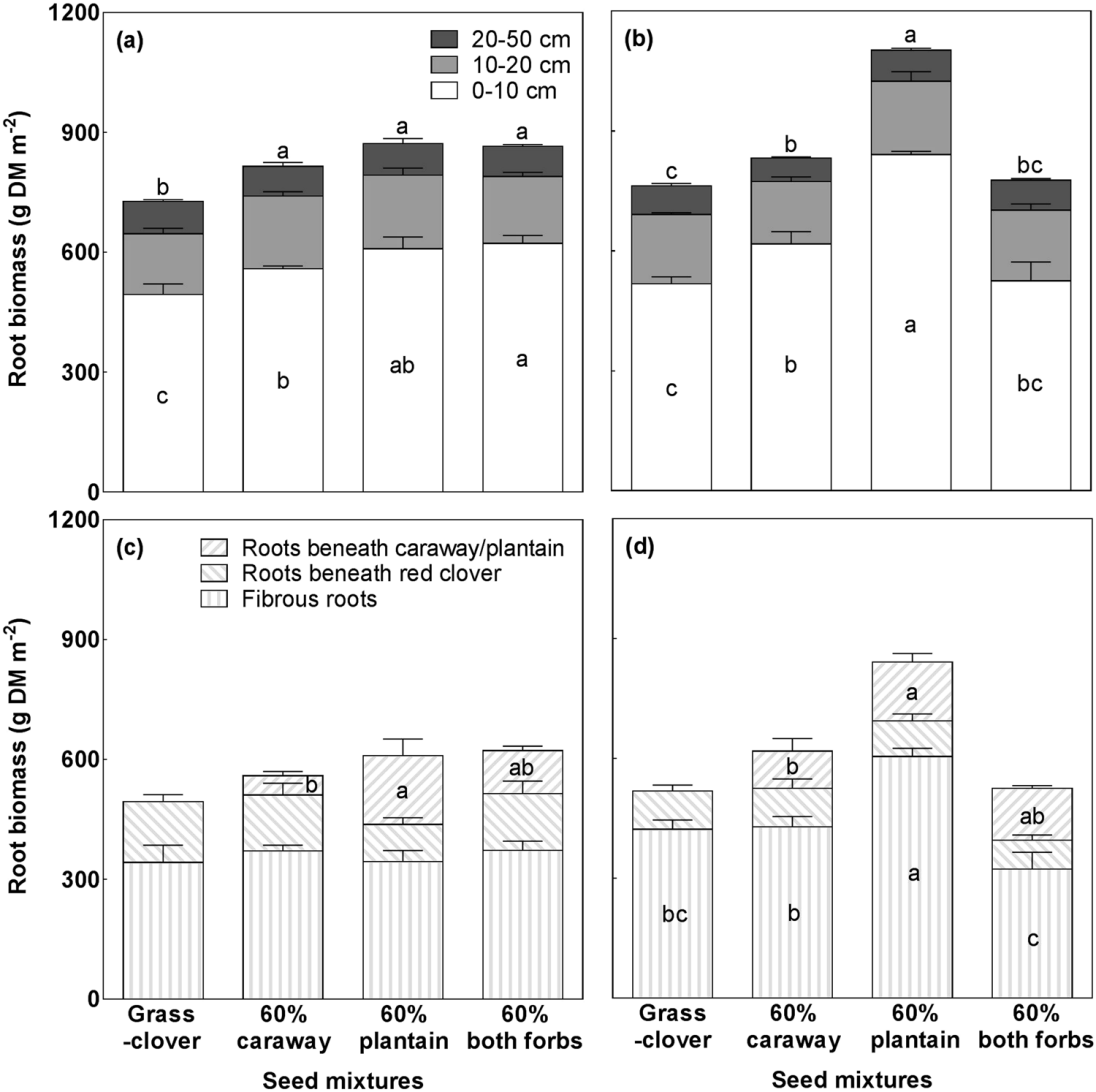
Standing root biomass in the four seed mixtures in August 2015. (**a**) at three soil depths without fertilisation; (**b**) at three soil depths with fertilisation; (**c**) for different root compartments at 0–10 cm depth without fertilisation; and (**d**) for different root compartments at 0–10 cm depth with fertilisation. Data are means ± SE (*n* = 3) for each soil depth or root compartment. Means for seed mixtures with different lowercase letters are significantly different (*P* < 0.05) using Tukey’s *post hoc* test.

Species composition and fertilisation interactively affected total root biomass down to 50 cm soil depth (Species composition × Fertilisation: *P* = 0.004; Fig. 4a,b). The unfertilised mixtures with 60% plantain or caraway or 60% of both forbs produced significantly more total root biomass than the grass-clover mixture (*P* = 0.01; Fig. 4a). Analysis of variance for the different soil layers showed that the increase in total root biomass occurred in the top 10 cm layer (*P* < 0.001), while no significant differences were found in the other two layers (*P* = 0.47 for 10–20 cm layer; *P* = 0.43 for 20–50 cm layer). We further analysed the root biomass in the top 10 cm layer at different sampling positions (either beneath red clover or the forb species where the soil core mainly comprised the taproots, or beneath ryegrass or between rows where fibrous roots constituted most of root biomass) to assess which root compartments were the main contributors to the increase in root biomass. Results showed that there were no significant differences in root biomass beneath ryegrass or between rows (*P* = 0.84), or beneath red clover (*P* = 0.45) between treatments (Fig. 4c). The larger root biomass in forb-containing mixtures than in the grass-clover mixture was mainly attributed to the root biomass of the forbs (*P* = 0.005).

Fertilisation resulted in significantly more root biomass in the mixture with 60% plantain than all other mixtures, with the increase also mainly occurring in the top 10 cm soil layer (*P* < 0.001; Fig. 4b). Analysis of root biomass at different sampling positions showed that the larger root biomass in the mixture with 60% plantain was mainly attributable to the larger biomass in the soil cores representing fibrous roots (*P* = 0.002; Fig. 4d).

Root biomass (0–10 cm depth) beneath plantain in the mixture with 60% plantain was significantly larger than that beneath the mixture with 60% caraway, regardless of fertilisation treatment (Fig. 4c,d). The results showed that root biomass per core was not significantly different between plantain and caraway, but the number of plants of plantain was significantly higher than that of caraway (Supplementary Figure 2), resulting in a larger total root biomass beneath plantain.

### Effects of species composition and fertilisation on bioenergy production and climate impact

**Figure 5:**
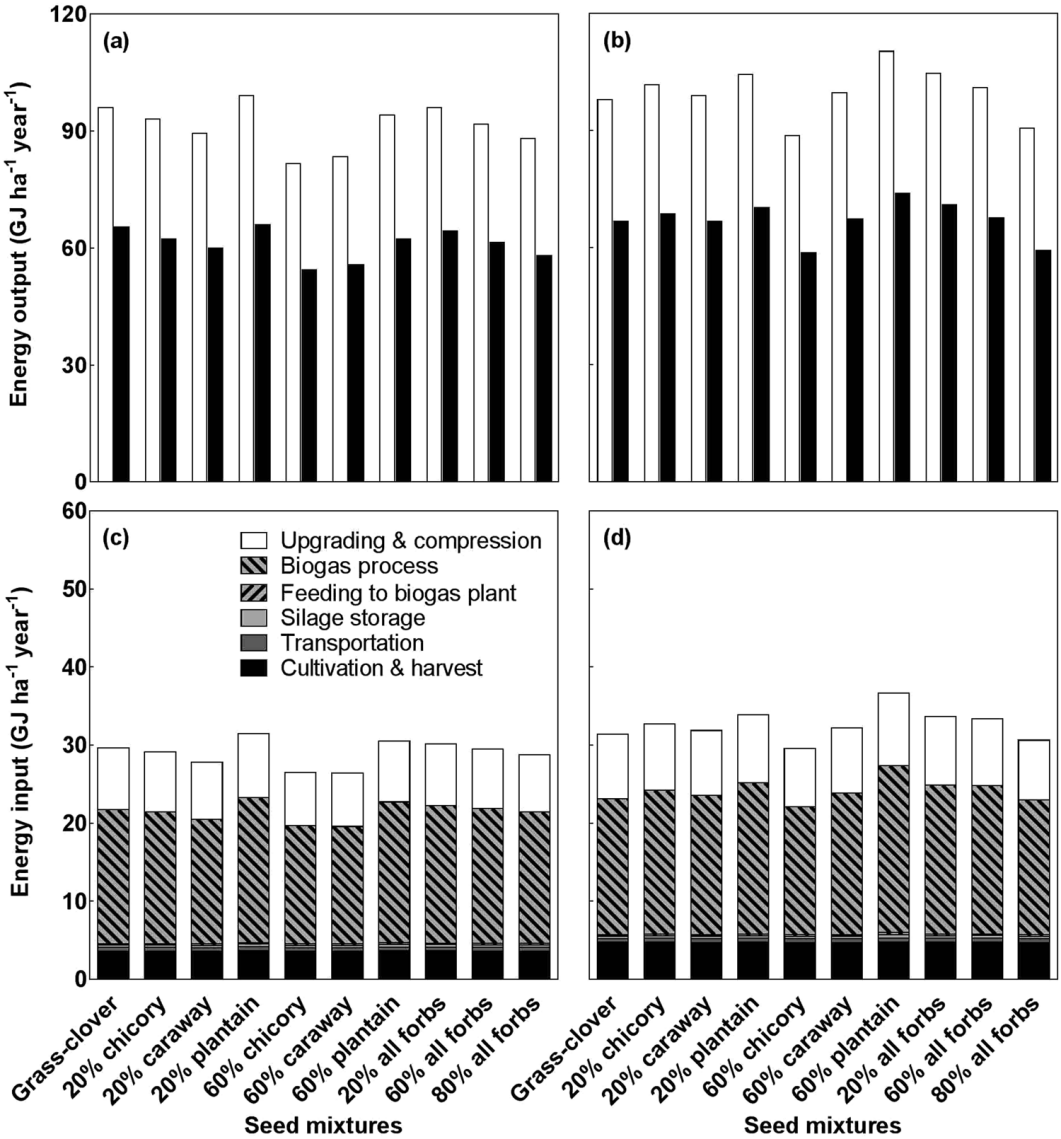
Energy output (**a**,**b**) and input (**c**,**d**) in the 10 seed mixtures without (**a,c**) and with fertilisation (**b**,**d**). Open and filled bars represent the methane energy output and the net energy balance, respectively **(a**,**b**). Different hatched bars denote various energy inputs (**c**,**d**). For detailed calculations of energy output and input, please see Supplementary Text and Table S1.

The methane energy output from the 10 mixtures ranged between 82 and 110 GJ ha^−1^ yr^−1^, with the 20% and 60% plantain-containing mixtures producing the highest energy yields both without and with fertilisation (Fig. 5a,b). The net energy balance ranging from 51 to 80 GJ ha^−1^ yr^−1^ across the mixtures showed a similar pattern as the energy output with the mixtures containing plantain producing 0.7 GJ ha^−1^ yr^−1^ and 7.3 GJ ha^−1^ yr^−1^ more than the grass-clover mixture without and with fertilisation, respectively. Fertilisation enhanced the methane energy output and the net energy balance both by 10%. Across mixtures and fertilisation treatments, the biogas process constituted the largest energy input (58% on average), followed by biogas upgrading and compression (26%), and cultivation and harvest (13%). The plantain-containing mixtures also had the highest energy requirements among all mixtures due to their large biomass that required more energy to process.

**Figure 6:**
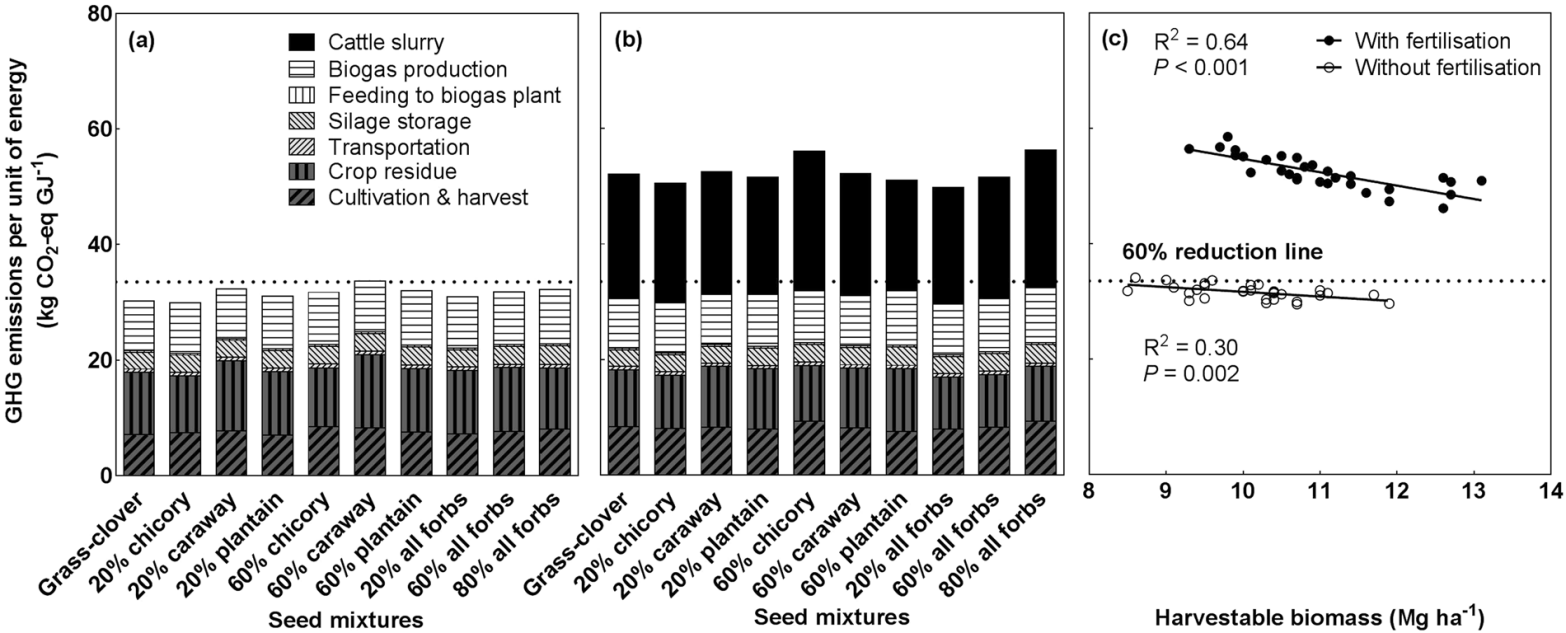
Greenhouse gas (GHG) emissions per energy unit in the 10 seed mixtures without (**a**) and with fertilisation (**b**), and plotted against harvestable biomass yields (**c**). The dashed horizontal line denotes 60% reduction in GHG emissions compared to fossil fuel. Different hatched bars denote various sources of GHG emissions (**a**,**b**). Significant linear regressions and coefficients of determination were shown (**c**). For detailed calculations of GHG emissions, please see Supplementary Text and Table S2.

Biomass from all unfertilised mixtures used for biogas production met the EU sustainability criteria (i.e. 60% reduction in GHG emissions per unit energy compared to fossil fuel) for renewable biofuels (Fig. 6a). Field N_2_O emissions from crop residues (35% on average), biogas production (29%) and cultivation and harvest (24%) made major contributions to GHG emissions of all sources, while silage storage (10%), transportation (2%) and feeding into the biogas plant (1%) were minor contributors to GHG emissions (Supplementary Figure S3). Biomass from all fertilised mixtures enhanced the amount of GHG emitted on average by 82% compared to unfertilised mixtures (Supplementary Figure S3), with fresh cattle slurry being the largest contributor. This resulted in none of the seed mixtures meeting the sustainability criteria (Fig. 6b). When plotting GHG emissions per unit of energy against harvestable biomass, it was found that GHG emissions significantly decreased with increasing biomass for all mixtures with slurry application (Fig. 6c). This was mainly because almost all GHG sources proportionally related their emissions to the biomass yield and therefore also to the energy yield. GHG emissions from unfertilised plots also showed a linearly decreasing pattern, but varied less across biomass yields compared to the fertilised plots.

## Discussion

We have demonstrated firstly, an increased above-ground productivity when including plantain in highly productive grass-clover mixtures thereby enhancing the bioenergy potential of the mixtures. Secondly, below-ground productivity was significantly increased when including plantain or caraway in the grass-clover mixtures implying a greater potential for soil C sequestration due to the presence of these forbs. Thirdly, the study showed that all unfertilised mixtures met the target of 60% reduction in GHG emissions for renewable biofuels, whereas fertilisation, although enhancing productivity and bioenergy production, resulted in increased GHG emissions of all mixtures reducing their potential to mitigate climate change.

The results showed that inclusion of plantain significantly improved total annual yields of the grass-clover mixture, whereas chicory and caraway maintained yields (Fig. 2). Higher yields of mixtures containing plantain were attributed to the high and stable yield of plantain as well as a stronger promotion of red clover yield by plantain (Fig. 3), most likely due to better above- and/or below-ground resource utilisation. Plantain has rosette leaves that hug the ground, allowing red clover to intercept more light. Below-ground, a greater proportion of plantain roots are branched secondary roots well distributed in the upper soil layers^14, 30^ allowing plantain access to more nutrients (e.g. available N) in the soil than chicory and caraway characterized by a larger proportion of thick tap-roots. The two other forbs in this study, chicory and caraway, did not improve yields of the grass-clover mixtures. In case of chicory this could be due to its high-statured broad leaves, which could to a larger extent have competed with red clover for light and thus, reducing clover growth. Caraway is known to have lower yields than the other two forbs^15, 31^, due to a greater investment in root development^32^. In summary, we found that inclusion of plantain in productive grass-clover mixtures further increased the yield potential of these grasslands, probably due to a better above- and below-ground resource utilization.

Inclusion of both plantain and caraway in the grass-clover mixture enhanced the standing root biomass (Fig. 4). For plantain this followed the increased above-ground productivity, whereas inclusion of caraway did not affect the above-ground yields of the grass-clover mixture. The larger root biomass beneath caraway and plantain was found mainly in the top 10 cm soil layer where the taproots of caraway and plantain contributed substantially to the greater root biomass of the mixtures. Hence, vertical niche differentiation between shallow-rooting and deep-rooting species^33^ does not seem prevailing in the present intensively managed grassland mixtures. Rather, our finding agrees with those of Mommer, *et al*.^34^ and Pirhofer-Walzl *et al*.^16^ showing that roots in mixtures tend to clump and increase competition for resources in the topsoil rather than being distributed over the whole profile. Fertilisation significantly enhanced the total root biomass of plantain-containing grass-clover mixtures with most of the increase occurring beneath grass and between rows in the top 10 cm soil layer. Thus, increased root growth of ryegrass upon fertilisation is a likely explanation for the positive fertilisation effect on the root biomass yields^35^. In the present study we did not measure changes in soil C pools since such investigation requires longer experimental duration than the present three years to be visible, nor did we estimate rhizodeposition, but the increased root biomass with inclusion of plantain and caraway strongly indicates an enhanced potential for soil C sequestration as root C is more important for soil C build-up than shoot C^36^.

The estimated methane energy output and net energy balances varied across seed mixtures with the plantain-containing grass-clover mixture having a higher energy yield than the other mixtures in accordance with our findings on productivity. This supports that biomass yields play a much more important role than specific methane yields in determining the area-specific methane yield^20, 22^. The methane energy output (82–110 GJ ha^−1^ yr^−1^) from the mixtures were similar to the 80 GJ ha^−1^ yr^−1^ reported in a Swedish study on temporary grass-clover mixtures^37^, whereas the net energy balance in the present study (51–80 GJ ha^−1^ yr^−1^) is higher than the 25 to 69 GJ ha^−1^ yr^−1^ reported for permanent grasslands^38–40^. Although fertilisation in accordance with previous studies^19, 22^ increased energy yields of the mixtures, in this case by 10%, it strongly weakened GHG mitigation in all the mixtures because of high field N_2_O emissions related to the added slurry. In fact, none of the fertilised mixtures met the EU sustainability criteria for renewable vehicle fuels. A recent soil incubation study showed that anaerobically digested cattle slurry lowered N_2_O emissions by 63% compared to fresh cattle slurry^41^ making this an option to improve the GHG balance for the fertilised mixtures.

The present results provide new evidence that biomass from unfertilised grass-clover mixture with inclusion of forbs holds a large potential for GHG mitigation when used as biogas feedstock. Thus, the findings from the present temporary grasslands corroborate with previous findings on marginal land^17–19^. Importantly, all unfertilised grass-clover-forb mixtures in the present study met the EU sustainability criteria for renewable biofuels, which provides an important incentive for developing bioenergy production systems based on low-input forb-containing grass-clover mixtures. In this study, the estimated field N_2_O emissions from crop residue ranged between 1.17 and 1.55 kg N_2_O-N ha^−1^ year^−1^ across the 10 seed mixtures, which agreed well with the field measured N_2_O emissions of 1.35 kg N_2_O-N ha^−1^ year^−1^ with standard error (0.07) from grass-clover mixtures conducted at the same site (Sørensen *et al*., unpublished data). Nevertheless, it should be stressed that the presently estimated GHG emissions were based on the single default emission factor for direct N_2_O emissions (i.e. 0.01 kg N_2_O-N kg^−1^ N). Previous studies have found that the larger biomass production associated with species diversity can reduce field N_2_O emissions through increased plant N uptake^42^ and consequently lower availability of soil mineral N for microbial N_2_O production. This implies that the forb-containing mixtures may have lower emission factors than the grass-clover mixture, which requires further experimental demonstration.

Recent studies have also shown that these competitive forbs generally contain higher mineral concentrations (such as P, Mg, K, S, Zn, B etc.) than grass and legumes^43^, whereas inclusion of such forbs in grass-clover mixtures lowered organic matter digestibility compared to grass-clover mixtures, thus reducing nutritive values of forages^44^. These results imply that increased biomass production and mineral concentrations due to inclusion of such forbs may not necessarily lead to the improvement of animal nutrition.

In conclusion, our findings show that the incorporation of competitive forbs such as plantain in grass-clover mixtures is a promising strategy for enhancing above- and below-ground primary production in temporary grasslands providing high-yielding renewable biomass for bioenergy production while contributing to climate change mitigation.

## Electronic supplementary material

Cong et al Supplementary Information
