# Supplementary material for: Forbs enhance productivity of unfertilised grass-clover leys and support low-carbon bioenergy
Source: Sci Rep. 2017 May 2;7:1422. doi: 10.1038/s41598-017-01632-4 (PMC5431050; doi:10.1038/s41598-017-01632-4)
Supplement: Supplementary file 1 — Cong et al Supplementary Information [file 41598_2017_1632_MOESM1_ESM.pdf]

Forbs enhance productivity of unfertilised grass-clover leys and support low-carbon bioenergy

Wen-Feng Cong[*], Jingying Jing, Jim Rasmussen, Karen Søegaard, and Jørgen Eriksen

Aarhus University, Department of Agroecology, Tjele, 8830, Denmark
[*]Corresponding author: wenfeng.cong@agro.au.dk

Supplementary Information

Net energy balance. The energy output as methane yield per hectare (ha) was calculated from harvestable biomass dry matter (DM) yields and specific methane yields. Harvestable DM yield was calculated from measured DM yields adjusted for the typically higher yield obtained using a Haldrup harvester than with full-scale machinery (Table S1). Biomass DM yield was further assumed to suffer a 5% loss during ensiling and feeding into the biogas plant[1]. Specific methane yields from forb-containing grass-clover (GC) mixtures were calculated according to previously determined specific methane yields (Nm^3 kg^{-1} VS) in batch tests and volatile solids (VS) content of GC mixtures and monocultures of chicory, caraway and plantain (Table S1). Given that co-digestion of crop residues of maize, wheat and perennial ryegrass was not found to produce significant synergistic or antagonistic effects on specific methane yields[2], here we assume the same methane production from individual components when digested alone or in mixtures. The resulting specific methane yields were further decreased by 10% to account for the lower methane production in a full-scale continuous biogas process than in a batch test[1]. The area-

specific methane yield was calculated separately for each of the four cuts since the specific methane yield and VS content change over the growing seasons[3].

The energy input consisted of the energy required for production of potassium fertilizer and lime, concrete bunker silo, plastic cover, biogas process, upgrading and compression as well as diesel consumption. Diesel was consumed for ploughing, harrowing, fertilization, liming, soil compaction, sowing and rolling using default values for a Danish context[4]. Diesel consumption used for cutting, chopping, transportation, compaction in the silo and feeding biomass into the biogas plant was calculated for each harvest occasion using different linear equations that related to harvestable biomass yield (Table S1). In this study, no pesticides were used. Cattle slurry was considered as animal waste (i.e. no energy required for producing cattle slurry). Seeds for establishing the perennial species were omitted because of minimal impact on the outcome. Other energy inputs including human labour and machinery were not included.

Greenhouse gas (GHG) emissions. Five main processes were considered to contribute to GHG emissions in this study, namely diesel consumption, production of lime and potassium fertilizer, silage storage, field nitrous oxide (N_2O) emissions from crop residues and cattle slurry, and losses during biogas production. Typical GHG emission factors were used to calculate GHG emissions from diesel consumption, lime and potassium fertilizer and silage storage (Table S2). Field N_2O emissions from above- and below-ground crop residues were estimated by multiplying the default emission factor for direct N_2O emissions from crop residue with the treatment-specific crop residue N. The amount of N in crop residues was calculated based upon harvested herbage biomass, standing root biomass, and N contents of harvested herbage and roots measured directly from this study. Indirect N_2O emissions from leached N that derived from crop

residues were estimated by multiplying the default emission factor from N leaching with the site-specific N leaching that was measured in a grassland experiment (next to this study) with the similar experimental setup and field managements. Direct and indirect N_2O emissions from cattle slurry were estimated from leached N and atmospheric re-deposited N and default emissions factors (Table S2). Cattle slurry was considered as animal waste in this study, i.e., with zero emission up to the point of collection. The soil C sequestration rate is not accounted for in the GHG balance according to the methodology described by the EU renewable energy directive[7]. The emissions from biogas production were calculated based upon an aggregated emission factor including emissions for heat and electricity use, the upgrading unit and the flare for excess biogas, methane losses from the biogas reactor, as well as direct and indirect emissions of N_2O from the digestate storage tank.

Table S1 Parameters used in the calculation of energy output and input for the production of biogas as a vehicle fuel based on biomass from the 10 seed mixtures in the multi-species grassland

Parameters	Unit	Values or equations	References
**Energy output**			
**Recovery coefficient**	%	74.603 + 1.3828 * biomass harvested by Haldrup (Mg DM ha^{-1})	Prade, *et al.*[8]
**DM loss during ensiling**	%	5	Gissén, *et al.*[1]
**Content of volatile solids**	Mg VS Mg^{-1} DM	Cut 1: GC[†] (0.91), CI (0.87), CA (0.88), PL (0.87)	Wahid, *et al.*[3]
		Cut 2: GC (0.93), CI (0.87), CA (0.91), PL (0.92)	
		Cut 3: GC (0.90), CI (0.88), CA (0.86), PL (0.90)	
		Cut 4: GC (0.90), CI (0.88), CA (0.87), PL (0.90)	
**Specific methane yield**	Nm^{3} Mg^{-1}VS[‡]	Cut 1: GC (0.352), CI (0.308), CA (0.348), PL (0.276)	Wahid, *et al.*[3]
		Cut 2: GC (0.342), CI (0.326), CA (0.279), PL (0.289)	
		Cut 3: GC (0.324), CI (0.299), CA (0.304), PL (0.273)	
		Cut 4: GC (0.338), CI (0.313), CA (0.302), PL (0.289)	
**Conversion efficiency[§]**	%	90	Gissén, *et al.*[1]
**Methane energy content**	MJ Nm^{-3}	35.3	
**Energy input**			
Cultivation			
**Spring ploughing**	L diesel ha^{-1}	20	Dalgaard, *et al.*[4]
**Light harrowing**	L diesel ha^{-1}	4	Dalgaard, *et al.*[4]
**Fertilization (cattle slurry)**	L diesel Mg^{-1} slurry	0.3	Dalgaard, *et al.*[4]
**Rate of cattle slurry**	Mg slurry ha^{-1} yr^{-1}	82.6	From this study
**Fertilization (potassium, K)**	L diesel ha^{-1}	2	Dalgaard, *et al.*[4]
**Production of potassium (K)**	MJ kg^{-1} K	7	Dalgaard, *et al.*[4]
**Potassium rate**	kg K ha^{-1}	200	From this study
**Liming**	L diesel ha^{-1} yr^{-1}	1.5	Dalgaard, *et al.*[4]
**Production of lime**	MJ Mg^{-1} lime	30	Dalgaard, *et al.*[4]
**Lime rate**	Mg lime ha^{-1} yr^{-1}	0.143	From this study
**Soil compaction**	L diesel ha^{-1}	2	Dalgaard, *et al.*[4]
**Sowing**	L diesel ha^{-1}	3	Dalgaard, *et al.*[4]
**Rolling**	L diesel ha^{-1}	2	Dalgaard, *et al.*[4]
**Diesel energy content**	MJ L^{-1} diesel	37.4	
Harvest			
**Cutting**	L diesel ha^{-1}	2.334 + 0.384 * biomass yield (0-17 Mg DM ha^{-1})	Prade, *et al.*[8]
		0.791 + 0.540 * biomass yield (>17 Mg DM ha^{-1})	
**Chopping**	L diesel ha^{-1}	11.072 + 0.268 * biomass yield (0-12 Mg DM ha^{-1})	Prade, *et al.*[8]

		1.203 * biomass yield (>12 Mg DM ha^{-1})	
Transportation	L diesel ha^{-1}	1.913 + 0.451 * biomass yield (0-12 Mg DM ha^{-1})	Prade, *et al.*[8]
		0.611 * biomass yield (>12 Mg DM ha^{-1})	
Silage storage			
**Ensiled biomass**	Mg fresh weight ha^{-1}	biomass yield (Mg DM ha^{-1})/DM content	From this study
**DM content of ensiled biomass**	%	35	Prade, *et al.*[8]
**Storage density of biomass**	Mg fresh weight m^{-3}	0.75	Prade, *et al.*[8]
**Energy required for silo**	MJ m^{-3} yr^{-1}	1.82	Prade, *et al.*[8]
**Energy required for plastic cover**	MJ m^{-3} yr^{-1}	6.4	Prade, *et al.*[8]
**Compaction in the silo**	L diesel ha^{-1}	0.343 * biomass yield (Mg DM ha^{-1})	Prade, *et al.*[8]
Feeding to biogas plant	L diesel ha^{-1}	0.189 * biomass yield (Mg DM ha^{-1})	Prade, *et al.*[8]
Biogas production			
**Energy required for heating**	MJ Mg^{-1} feedstock	110	Berglund & Börjesson[9]
**Energy required for electricity**	MJ Mg^{-1} feedstock	66	Berglund & Börjesson[9]
**Biomass feedstock**	Mg feedstock ha^{-1}	biomass yield (Mg DM ha^{-1})/DM content	From this study
**DM content of feedstock**	%	10	Berglund & Börjesson[9]
Biogas upgrading			
**Energy required for upgrading**	MJ Nm^{-3}	1.08	Prade, *et al.*[8]
**Biogas volume**	Nm^{3} Mg^{-1}VS	Cut 1: GC (0.657), CI (0.583), CA (0.653), PL (0.528)	Wahid, *et al.*[3]
		Cut 2: GC (0.636), CI (0.636), CA (0.518), PL (0.546)	
		Cut 3: GC (0.593), CI (0.583), CA (0.618), PL (0.516)	
		Cut 4: GC (0.622), CI (0.614), CA (0.601), PL (0.555)	
**Conversion efficiency**[§]	%	90	Gissén, *et al.*[1]
Methane compression			
**Energy required for upgrading**	MJ Nm^{-3}	0.72	Prade, *et al.*[8]
**Conversion efficiency**[§]	%	90	Gissén, *et al.*[1]

[†] GC refers to grass-clover mixture; CI, chicory; CA, caraway; PL, plantain.
[‡] Nm^{3} refers to methane volume normalised to standard conditions (0 °C and 1.013 bar).
[§] Conversion efficiency refers to % of methane yield determined at laboratory scale that is achieved in a full-scale continuous biogas process.

Table S2 Parameters used in the calculation of GHG emissions from the production of biogas as a vehicle fuel based on biomass from the 10 seed mixtures in the multi-species grassland

**Parameters**	**Unit**	**Values or equations**	**References**
Diesel consumption[†]			
**Diesel GHG emission factor**	g CO_2-eq MJ^{-1}	83.8	European Union[7]
Liming			
**Direct CO_2 emission factor**	kg CO_2-eq kg^{-1} lime	0.44	IPCC[5]
**Indirect CO_2 emission related to excavation and transport**	kg CO_2-eq kg^{-1} lime	0.019	Elsgaard, *et al.*[10]
Potassium (K) fertilizer			
**CO_2 emission factor**	kg CO_2-eq kg^{-1} K	0.46	LowCVP[11]
Silage storage			
**Plastic cover**	g CO_2-eq MJ^{-1}	72	Börjesson, *et al.*[12]
**Bunker silo**	g CO_2-eq MJ^{-1}	67.9	Börjesson, *et al.*[12]
N_2O emissions from crop residue			
**Belowground to aboveground ratio**[‡]	-	Without slurry: GC (0.36), 60CA (0.45), 60PL (0.41), 60CCP (0.42)<br>With slurry: GC (0.37), 60CA (0.39), 60PL (0.43), 60CCP (0.34)	From this study
**Aboveground residues as share of harvested biomass**	-	0.16+0.84/biomass yield	Prade, *et al.*[8]
**N content of aboveground biomass**[*]	g N g^{-1} DM	Without slurry: GC (0.030), 20CI (0.024), 20CA (0.029), 20PL (0.026), 60CI (0.023), 60CA (0.027), 60PL (0.025), 20CCP (0.025), 60CCP (0.024), 80CCP (0.021)<br>With slurry: GC (0.027), 20CI (0.023), 20CA (0.026), 20PL (0.025), 60CI (0.023), 60CA (0.024), 60PL (0.026), 20CCP (0.024), 60CCP (0.022), 80CCP (0.022)	Dhamala, *et al.* submitted
**N content of belowground biomass**[*]	g N g^{-1} DM	Without slurry: GC (0.019), 20CI (0.018), 20CA (0.019), 20PL (0.018), 60CI (0.017), 60CA (0.019), 60PL (0.016), 20CCP (0.018), 60CCP (0.017), 80CCP (0.016)<br>With slurry: GC (0.017), 20CI (0.017), 20CA (0.018), 20PL (0.017), 60CI (0.016), 60CA (0.019), 60PL (0.016), 20CCP (0.017), 60CCP (0.017), 80CCP (0.016)	Dhamala, *et al.* submitted

**Direct N_2O emission factor for crop residues**	kg N_2O-N kg^{-1} N	0.01	IPCC[5]
**N leaching from crop residue[§]**	kg N ha^{-1}	14	Eriksen, *et al.*[13]
**Indirect N_2O emission factor from leaching**	kg N_2O-N kg^{-1} leached N	0.0075	IPCC[5]
**N_2O global warming potential**	kg CO_2-eq kg^{-1} N_2O	298	IPCC[5]
N_2O emissions from cattle slurry			
**Direct N_2O emission factor for cattle slurry**	kg N_2O-N kg^{-1} N	0.01	IPCC[5]
**Fraction of N volatilization from cattle slurry**	kg N kg^{-1} N	0.20	IPCC[5]
**Indirect N_2O emission factor for N re-deposition from cattle slurry**	kg N_2O-N kg^{-1} N	0.01	IPCC[5]
**N leaching from cattle slurry[§]**	kg N ha^{-1}	3	Eriksen, *et al.*[13]
**Indirect N_2O emission factor for N leaching from cattle slurry**	kg N_2O-N kg^{-1} N	0.0075	IPCC[5]
Biogas production[‖]			
**Aggregated emission factor**	kg CO_2-eq Mg^{-1} DM	54.3	Björnsson[6]

[†] Diesel consumption includes diesel for cultivation and harvest (ploughing, harrowing, fertilization, liming, soil compaction, sowing, rolling, cutting, chopping), transportation, compaction of ensiled biomass and feeding to biogas plant.

[‡] GC refers to grass-clover mixture; CI, chicory; CA, caraway; PL, plantain; 20CI, 20CA, 20PL, 60CI, 60CA, 60PL refer to 20% or 60% chicory or caraway or plantain grown in the grass-clover mixture; 20CCP, 60CCP, 80CCP refers to 20%, 60%, 80% of the three forbs in the grass-clover mixture. Belowground to aboveground ratio of GC, 60CA, 60PL or 60CPP was calculated by dividing respective root biomass sampled in August 2015 by cumulative harvested biomass over the two years. These belowground to aboveground ratios were used to calculate root biomass from the other mixtures, assuming that R/S ratio did not significantly vary with proportion of forbs in the mixture. For example, we assume that the mixture containing 60% caraway had the same R/S ratio as the mixture containing 20% caraway.

[*] N content of aboveground biomass (herbage) and belowground biomass (roots) were measured for each of the 10 seed mixtures. The data came from a PhD study conducted in this same field experiment (Dhamala et al. submitted).

[§] The data of N leaching were from a grassland experiment (just next to our experiment) with very similar experimental set-up and field managements, where grass-clover leys were grown subjected to four cuts with or without slurry application (200 kg total-N ha^{-1}).

[‖] Biogas production is an aggregated emission factor including emissions for heat and electricity requirements, methane losses from the biogas reactor, the upgrading unit and the flaring of excess biogas as well as direct and indirect emissions of nitrous oxide (N_2O) from the digestate storage tank.

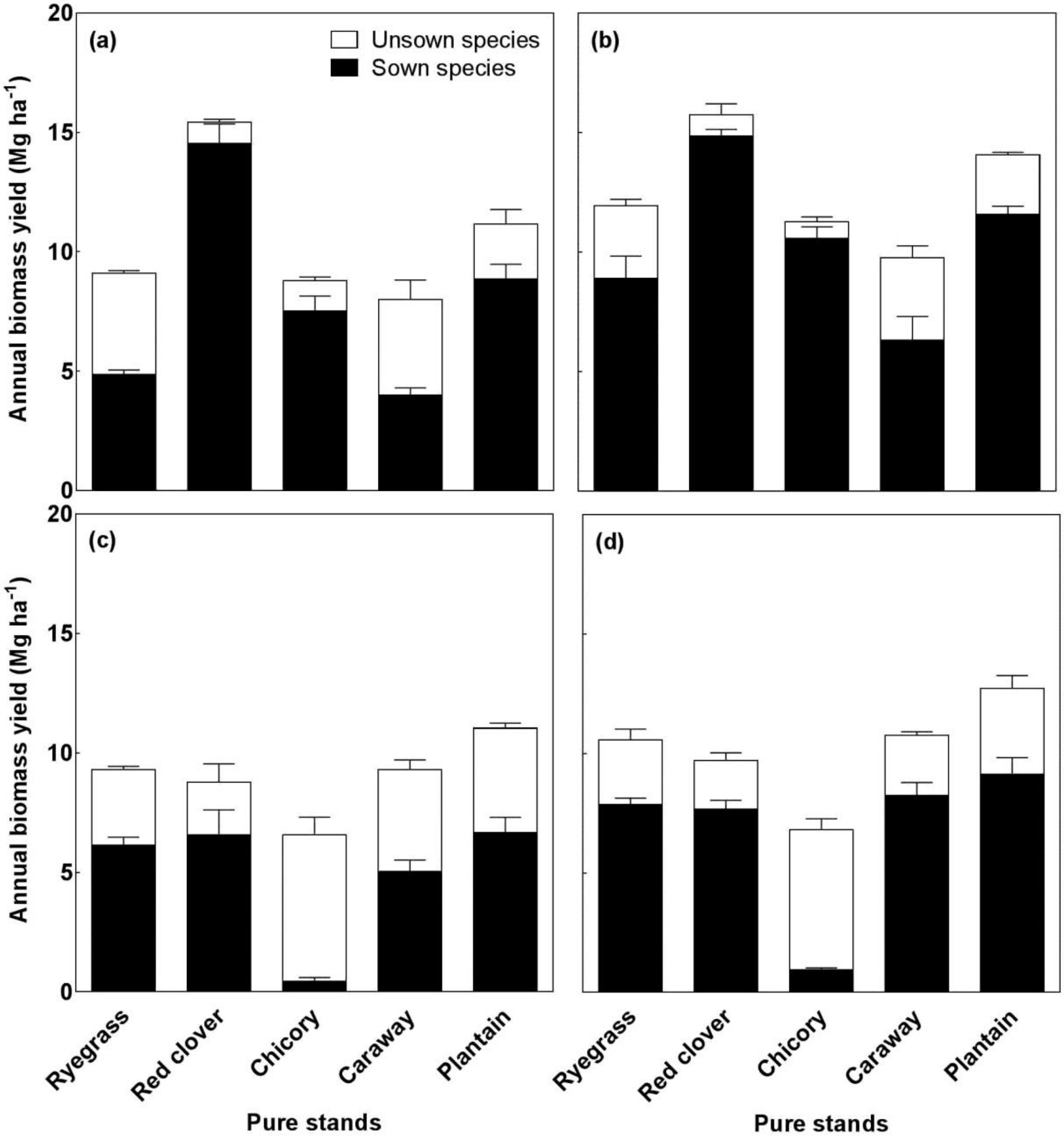

Figure S1. Annual biomass yield of five pure stands in 2014 (a,b) and 2015 (c,d) without (a,c) and with (b,d) fertilisation. Data are means ± SE (*n* = 3) for sown and unsown species, respectively.

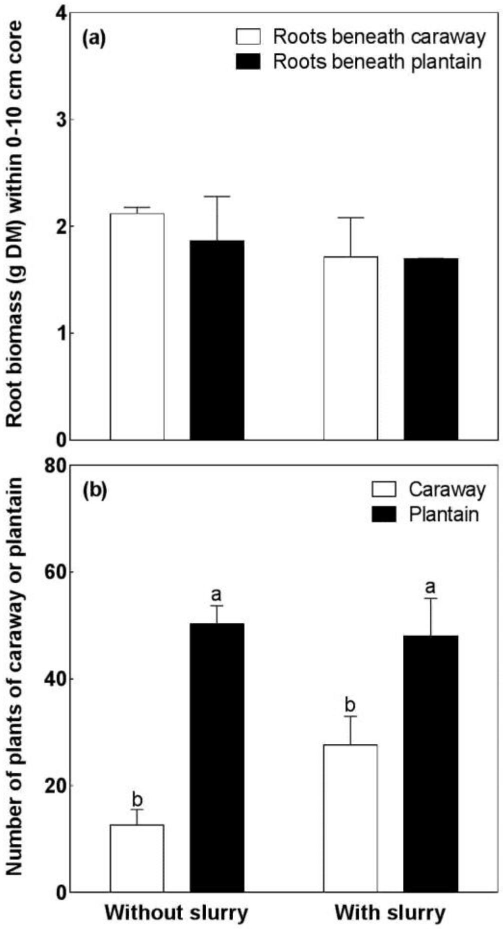

Figure S2. Standing root biomass within the core (0-10 cm depth) taken on individual plants of caraway or plantain (a) and the number of plants of caraway or plantain in the sampling area (b) taken in late August 2015 in caraway- and plantain-containing grass-clover mixtures with and without fertilisation. Data are means ± SE (n = 3). Means with different lowercase letters show significant differences ($P < 0.05$) between caraway and plantain using Tukey's *post hoc* test.

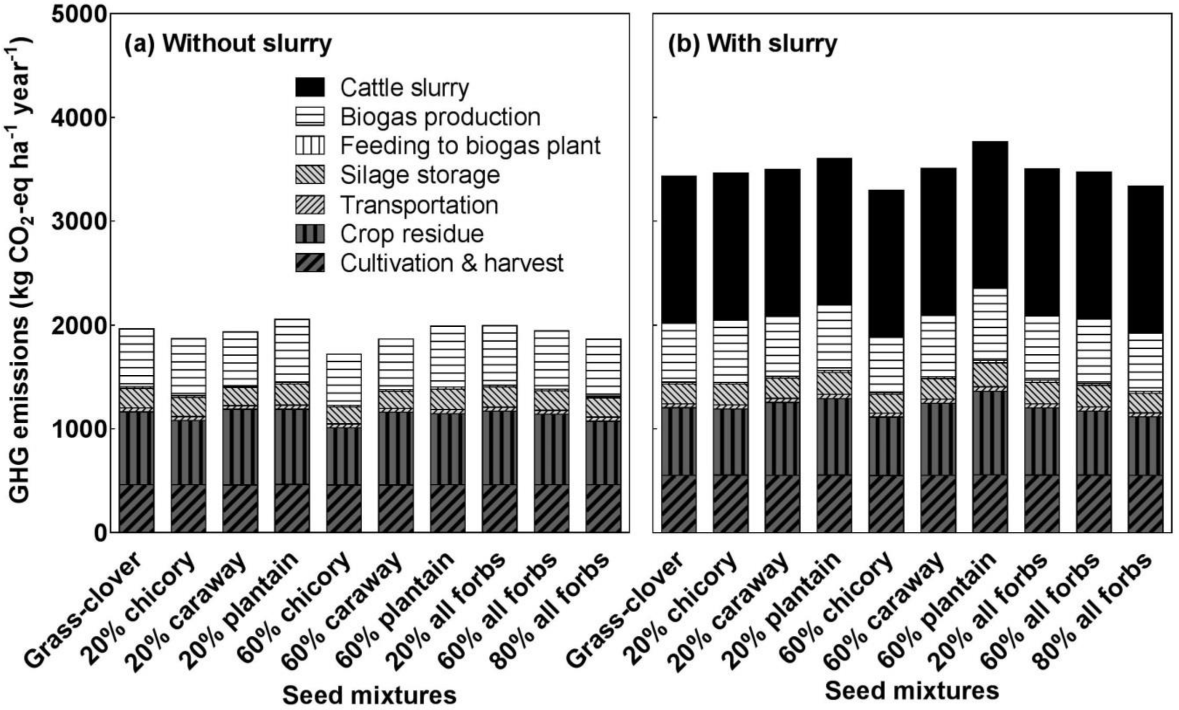

Figure S3. Greenhouse gas (GHG) emissions throughout the production process in the 10 seed mixtures without (a) and with fertilisation (b). Different hatched bars denote all sources of GHG emissions. For detailed calculations of GHG emissions, please see Supplementary Text and Table S2.

References

1. Gissén, C. *et al*. Comparing energy crops for biogas production – Yields, energy input and costs in cultivation using digestate and mineral fertilisation. *Biomass Bioenergy* **64**, 199-210 (2014).
2. Jahn, L. *et al*. Can specific methane yield be enhanced by co-fermenting different crop substrates? *Grassl. Sci. Eur.* **17**, 469-471 (2008).
3. Wahid, R., Ward, A. J., Moller, H. B., Soegaard, K. & Eriksen, J. Biogas potential from forbs and grass-clover mixture with the application of near infrared spectroscopy. *Bioresour. Technol.* **198**, 124-132 (2015).
4. Dalgaard, T., Halberg, N. & Porter, J. R. A model for fossil energy use in Danish agriculture used to compare organic and conventional farming. *Agric. Ecosyst. Environ.* **87**, 51-65 (2001).
5. IPCC. *IPCC Guidelines for National Greenhouse Gas Inventories. 4* (ed. Eggleston, H.S., Buendia, L., Miwa, K., Ngara, T. & Tanabe, K.) (Hayama, 2006).
6. Björnsson, L. *Energigrödor för biogasproduktion - Del 3 - Energi- och växthusgaseffektivitet*. (Lunds University, Lund, 2013).
7. European Union. Directive 2009/28/EC of the European Parliament and of the Council. *OJ* **140**, 16-62 (2009).
8. Prade, T., Svensson. S-E., Hörndahl, T., Kreuger, E. & Mattsson, J. E. *Grass-clover ley and whole-crop cereals as biogas substrate – Evaluation of influence of harvest date and cutting length on energy yield and substrate costs*. (Swedish University of Agricutltural Sciences, Alnarp, 2015).
9. Berglund, M. & Börjesson, P. Assessment of energy performance in the life-cycle of biogas production. *Biomass Bioenergy* **30**, 254-266 (2006).
10. Elsgaard, L., Olesen, J. E., Hermansen, J. E., Kristensen, I. T. & Børgesen, C. D. Regional greenhouse gas emissions from cultivation of winter wheat and winter rapeseed for biofuels in Denmark. *Acta Agri. Scandinavica Section B Soil Plant Sci.* **63**, 219-230 (2013).
11. LowCVP. Well-to-wheel evaluation for production of ethanol from wheat. A Report by the LowCVP Fuels Working Group, WTW Sub-Group. FWG-P-04-024 (2004).
12. Börjesson, P., Tufvesson, L. & Lantz, M. *Livscykelanalys av svenska biodrivmedel.* (Lunds University, Lund, 2010).
13. Eriksen, J., Askegaard, M., Rasmussen, J. & Søegaard, K. Nitrate leaching and residual effect in dairy crop rotations with grass–clover leys as influenced by sward age, grazing, cutting and fertilizer regimes. *Agric. Ecosyst. Environ*. **212**, 75-84 (2015).
